# Telehealth Utilization During the COVID-19 Pandemic: A Preliminary Selective Review

**DOI:** 10.1089/tmr.2021.0040

**Published:** 2022-02-03

**Authors:** Amelia Harju, Jonathan Neufeld

**Affiliations:** Institute for Health Informatics, University of Minnesota, Minneapolis, Minnesota, USA.

**Keywords:** telehealth, telemedicine, COVID-19, pandemic, utilization, disparities

## Abstract

**Background:** The COVID-19 pandemic reduced in-person visit volume and fueled a corresponding explosion in demand for telehealth services, resulting in the enactment of several temporary state and federal policies to allow greater flexibility in delivering telehealth services. This review examines patterns in telehealth utilization during the pandemic by synthesizing available findings from large-scale studies.

**Methods:** To be included in this review, studies must be of original research, include data from 2020 or 2021, have a U.S. study population, and analyze telehealth encounter data across multiple payers and health systems. This review includes 10 studies that fully met the inclusion criteria and 29 studies that examined telehealth use during the pandemic, although not from multipayer, multihealth system data sets. All studies were identified using Ovid MEDLINE and Google Scholar.

**Results:** At its peak, telehealth accounted for roughly 15–50% of visits across the various studied populations and data sets. The more telehealth was utilized, the smaller the decrease in overall visit volume. Audio visits tended to be used more often than video visits, and telehealth utilization varied across geographic regions and medical specialties. There were disparities in telehealth use by race, age, income, and other factors.

**Discussion:** Most telehealth visits during the pandemic would not have been reimbursable without the telehealth policy changes that took place. The variability in telehealth utilization across geographic regions is likely attributed to state-level telehealth policies. Most studies examining disparities in telehealth utilization did not compare disparities from before and during the pandemic, and these disparities may be a characteristic of health care overall rather than of telehealth specifically.

## Introduction

### Background

The impact of the COVID-19 pandemic on the delivery of health care services in the United States was rapid and profound, reducing in-person visit volume due to travel and contact restrictions and fueling a corresponding explosion in demand for services that could be delivered remotely to patients outside the clinic setting. In response to this rise in demand, several temporary policies were enacted at state and federal levels to allow greater flexibility in delivering health care services via telehealth during the public health emergency (PHE).^1^

The resulting surge in telehealth utilization has been reported in several narrative collections^2–4^ and left a huge volume of telehealth claims data that have started to be analyzed almost as soon as they became available. Several researchers have published studies using large data sets to explore this surge from a variety of perspectives. Although none of these studies can claim to be either representative of, or definitive for, telehealth as a whole, we believe it is now possible to begin to categorize, summarize, and glean common themes and findings that are starting to emerge.

We undertook this review to begin to synthesize findings available from the large-scale studies published thus far, and also to lay some methodological groundwork for the interpretation of these and any future findings related to telehealth utilization patterns during the pandemic.

#### Telehealth policies during the PHE

During the early stages of the COVID-19-related PHE, state and federal governments approved a series of temporary rules and waivers designed to allow greater use of telehealth to support the continuation of health care services in the context of widespread travel restrictions and efforts to reduce transmission of the virus.^1^ Some of the most relevant and impactful PHE-related telehealth policy changes included the following flexibilities granted by the Department of Health and Human Services^1^

● Increased flexibility for telehealth licensing and interstate compacts, making it easier for health care providers to deliver telehealth services across state lines.
● Altered federal policies for Medicaid and Medicare that:
○ Allowed providers to deliver telehealth services to both new and established patients, patients who are located in their home, and patients who live outside of designated rural areas.
○ Expanded the list of health care services that can be delivered via telehealth, especially for audio visits.
○ Reimbursed audio visits and video visits at the same rate as in-person services.
● Temporarily suspended enforcement of HIPAA requirements, allowing a greater variety of virtual platforms to be used for telehealth visits.
● Temporarily allowed providers to prescribe some controlled substances via telehealth without requiring an in-person visit.

Commercial payers followed suit (usually compelled by state emergency mandates), leading to near-universal third-party coverage for the full range of telehealth services.^5^ As a result of these policies, health care facilities were able to rapidly transition to providing services via telehealth in an effort to maintain access to care and compensate for reductions in in-person visit volume. Moreover, the potential services implemented included, for the first time, the full range of technically feasible virtual services. Not all services were reimbursed equally at first, and the type and amount of reimbursement ultimately may have driven utilization in significant ways.^5,6^ Nevertheless, the PHE has unquestionably yielded the richest telehealth claims data sets that have ever been available to telehealth services researchers.

### Purpose

Several large-scale claims studies have now been published examining telehealth utilization patterns in the United States during the first year of the pandemic. In this article we begin synthesizing these reports to determine what common findings, themes, and principles, if any, can be gleaned from them. This will no doubt be one of many such syntheses, as the research community continues to explore and unpack these data over the next several years or more.

This process can help determine the impact of PHE-related telehealth policies on different types of telehealth use (e.g., audio visits, video visits, e-consults), determine the extent that telehealth was able to offset declines in in-person visits, compare telehealth use across different specialties and diagnoses, examine geographical variation in telehealth use, and examine disparities in telehealth use (relative to disparities in overall health care utilization).

This review synthesizes information from 10 articles that analyzed large-scale, multipayer, multihealth system data sets to examine patterns in telehealth utilization in the United States during the COVID-19 pandemic and explore the ways in which the findings from these studies varied. This review also briefly summarizes information from an additional 29 articles that examined telehealth use during the pandemic, although not from multipayer, multihealth system data sets.

## Methods

We identified studies for review using Ovid MEDLINE and Google Scholar. The inclusion criteria for the initial search were as follows:

● Original research (i.e., not a review, commentary)
● Study population exclusively in the United States
● Includes data from 2020 or 2021
● Includes telehealth utilization data that are highly “generalizable”:
○ Includes telehealth utilization data from electronic health records (EHR) or claims data, AND
○ Data include multiple payers, states, provider types, and health systems.

The Ovid MEDLINE search was conducted on June 21, 2021, and used the following MeSH words as search terms: telehealth; Medicare; Medicaid; health benefit plans, employee; insurance, health; delivery of health care; and COVID-19. The MeSH words that are directly related or synonymous were combined with “or,” resulting in the following search: telehealth AND COVID-19 AND delivery of health care AND (Medicare OR Medicaid OR health benefit plans, employee OR insurance, health). This search produced 137 articles. Of these articles, three were selected for the primary analysis for this review based on the inclusion criteria described above, and 11 were included in this review for a peripheral analysis.

Twenty articles were excluded due to not being original research, nine articles were excluded due to the study population not being exclusively in the United States, three articles were excluded due to the data analyzed not being from 2020 or 2021, and 91 articles were excluded due to not including an analysis of telehealth EHR or claims data.

The Google Scholar search was conducted on June 24, 2021, and used the following search terms: telehealth utilization trends COVID-19. The date range was set to 2020–2021. This search produced 5,690 articles. Articles from the first 10 pages (i.e., the first 100 articles) were considered for inclusion in this review.

Of these 100 articles, seven were selected for the primary analysis of this review based on the inclusion criteria, and 17 were included in this review for a secondary analysis. Eleven articles were excluded due to not being original research, nine articles were excluded due to the study population not being exclusively in the United States, two articles were excluded due to the data analyzed not being from 2020 or 2021, and 54 articles were excluded due to not including analysis of telehealth EHR or claims data.

There are two additional articles from The Commonwealth Fund that did not show up in either search but are included in the primary analysis of this review due to meeting the inclusion criteria. There is also one additional article from the Office of the Assistant Secretary for Planning and Evaluation (ASPE) and one additional article from the Medicare Payment Advisory Commission (MEDPAC) that did not show up in either search but are included in the secondary analysis of this review due to their quality and relevance.

**FIG. 1.**
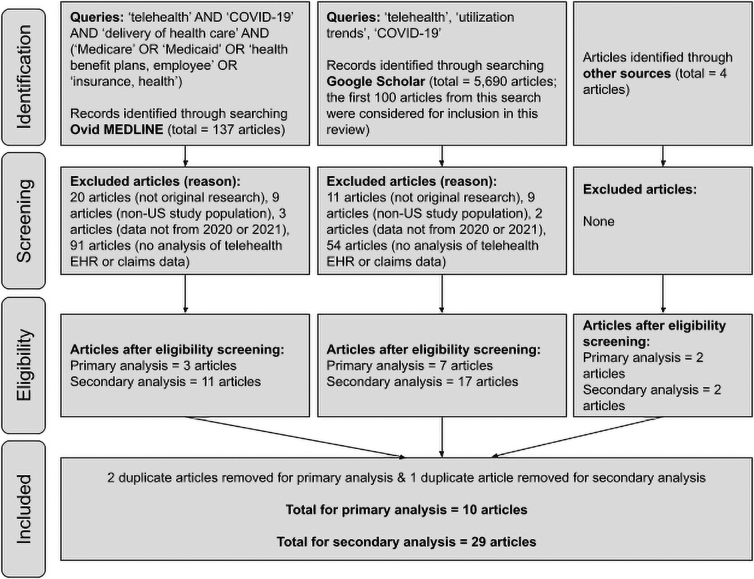
Summary of the literature review selection process.

After removing duplicates, 10 articles were included in the primary analysis for this review and 29 articles were included in the secondary analysis. A summary of these articles is presented in Supplementary Table S1, and a summary of the selection process for this review is displayed in Figure 1.

### Methodological considerations

A few preliminary points warrant mentioning at the outset. First, none of these studies can claim to be a representative sample of telehealth services in general. Each is a convenience sample or case series of sorts, even though some are quite large and diverse. Because telehealth services (such as all health services) are systematically influenced by reimbursement and other policy factors, it is impossible to observe telehealth utilization in the abstract. Although these large data sets may be highly informative, and may even cover entire universes of cases for a given set of payers or providers, none can represent “telehealth” in an unbiased way. Consequently, we avoid using the term “sample” and exercise caution to avoid unjustifiable generalizations when reviewing these findings.

Second, most of these studies only partially differentiate telehealth modalities (if at all). Indeed, during the early weeks of the pandemic, claims procedures were in flux and policies could change from week to week. All claims data are subject to coding errors, and it is only reasonable to expect a significant degree of errors during time periods in which billing policies are unclear or unstable. Most studies we reviewed did not differentiate live video from audio-only encounters, and portal-based or store-and-forward services were treated in a variety of ways. We can hope to gather a general sense of the overall volume of telehealth services from reviewing these studies, but a more exact understanding will require greater rigor.

Finally, we will use the terms “video” for services that use live, interactive two-way videoconferencing and “audio” for audio-only or telephonic services. In our estimation, these simplifications improve the clarity and readability of the analysis without any loss of specificity.

## Results

### Large-scale, multipayer, multihealth system data sets

Ten studies that analyzed large-scale, multipayer, multihealth system data sets met the selection criteria to be included in this review. All of these studies showed that in-person visits decreased dramatically during the early months of the pandemic, while telehealth use simultaneously increased across various specialties, peaking in March–April 2020.^7–16^ After the peak, telehealth use slowly declined through October 2020 (still remaining much higher than the prepandemic baseline). Mehrotra et al. observed that telehealth increased again before the year's end, in November and December, among various different types of provider organizations across the United States.^10^

About a third of provider organizations never used telehealth during 2020, while many organizations went from delivering 5% or more of services via telehealth to <5% from April to September.^9^ These researchers also found that larger organizations delivered a higher percentage of their visits via telehealth than organizations with fewer providers.

Two studies reported that telehealth utilization partially, but not fully, offset decreases in the total visit volume, ranging from 14% of total outpatient encounters to 48% of total outpatient encounters during April and May 2020.^7,15^ In general, the more telehealth was used, the smaller the decrease in visit volume during the pandemic.^8,13,15,16^ One study indicated a higher video visit volume relative to audio visits, but no other studies examined differences by telehealth modality.^14^

Telehealth use varied widely across specialties, with very low utilization for ophthalmology, rehabilitation, and surgical visits and high utilization for behavioral health, endocrinology, and neurology visits.^9,10,13,14^ Telehealth use also varied across diagnoses, ranging from 3% of visits for glaucoma to 53% of visits for depression.^13^ Behavioral health outpatient visits were the most likely form of specialty care to be delivered via telehealth, and as a result, the total visit volume for behavioral health declined less than other types of outpatient visits.^10,13,14,16^

Telehealth use was found to vary by geographic region, but results were mixed and none of the studies examined regional variation as distinct from state-based variation. Higher telehealth use was associated with higher regional COVID-19 prevalence during the beginning of the pandemic, but this association diminished after the first month of the pandemic.^7,11,14^ One study found that telehealth use ranged from 8% of all visits in South Dakota to 48% in Massachusetts by May–June 2020, and that states in the South tended to have lower telehealth utilization compared with other parts of the country.^12^ A different study showed that telehealth use ranged from 15% of all visits in the East North Central region to 27% in the Pacific region.^7^

Disparities in telehealth utilization were reported related to urbanicity, race/ethnicity, income/poverty, access to other resources, and age.^8,11,13–15^ Researchers found telehealth use was higher for patients with more social resources,^14^ greater disease burdens,^14^ and for young and middle-aged adults compared with children and older adults.^8^ Telehealth use was also found to be higher in urban areas^8,11^ and areas with fewer people of color,^15^ lower poverty levels,^8,11,13,15^ greater broadband availability,^11^ and higher prepandemic telehealth utilization.^11^ One study showed similar telehealth utilization rates among white and black patients.^7^

### Limited data sets

#### Single-payer studies

Three studies that analyzed single-payer data met the selection criteria to be included in the secondary analysis of this review. One study found that telehealth encounters among four major telehealth platforms (Amwell, Teladoc, MDLIVE, and Doctor on Demand) increased by 50% from the first quarter of 2019 to the first quarter of 2020, peaking at a 154% increase in visits at the end of March 2020.^17^ The portion of visits that were related to COVID-19 significantly increased from 6% to 16% in March 2020.^17^

The other two studies found that Medicare fee-for-service (FFS) telehealth encounters in 2020 increased greatly from February to April—which partially compensated for the decrease in in-person services—then started declining as in-person visits rebounded in mid-April and May, and finally leveled off by June.^18,19^ From January to June 2020, about 78% of telehealth encounters among Medicare FFS beneficiaries were primary care visits, and about one-third of these visits were audio Evaluation and Management (E&M) services.^19^ Bosworth et al. found that urban areas tended to have larger increases in Medicare primary care telehealth utilization than rural areas.^18^

#### Veterans health administration studies

Three studies that analyzed Veterans Health Administration (VA) EHR data met the selection criteria to be included in the secondary analysis of this review. Similar to results from other studies, reports using VA data showed that in-person visits decreased during the pandemic, while telehealth utilization increased dramatically.^20–22^ Sites that offered telehealth services before the pandemic had lower reductions in overall visit volume compared with sites that did not offer telehealth before the pandemic.^20^ All three studies indicated a higher audio visit utilization relative to video visits.^20–22^ Behavioral health outpatient visits were most likely to be delivered via telehealth, and as a result, the total visit volume for behavioral health declined less than other types of outpatient visits.^21^

Similar to results from other types of studies, telehealth utilization during the pandemic was higher among veterans who lived in urban areas, had higher disease burdens, and who were young and middle-aged adults relative to older adults.^21^ However, there was a different pattern in telehealth utilization regarding incomes, where veterans with lower incomes were actually more likely to receive telehealth services compared with those with higher incomes.^21^ In addition, telehealth use was lower among unhoused veterans.^21^

#### Single health system studies

Sixteen studies that analyzed EHR data from a single non-VA health system met the selection criteria to be included in the secondary analysis of this review. The results of these studies showed patterns similar to those from other types of studies. All studies indicate a decrease in in-person visit volume and a dramatic increase in telehealth visit volume across various specialties during the pandemic, generally peaking in March–April 2020.^23–38^ The increase in telehealth utilization partially offset the decline in in-person visit volume for oncology, inpatient, emergency department, and nonbehavioral health visits.^32,36,37^ Some studies observed that the increase in telehealth utilization fully offset the decline in total outpatient^36^ and behavioral health visits^30,37^ in their health systems.

Several studies analyzed results by telehealth modality. Five studies indicated that audio visits were used more often than video visits,^24,30–32,38^ whereas one study indicated the opposite.^33^ Two studies found similar utilization for audio visits and video visits,^23,34^ and one study found audio visits predominating in March–April 2020, and then, video visits predominating by May 2020.^37^ Two studies were at health systems that offered audio visits only in the event of technical issues that prevented a video visit from occurring.^28,29^

Telehealth use increased dramatically across many different specialties and patient diagnoses, including urgent care, cancer care, dermatology, pregnancy-related ambulatory visits, and chronic illnesses.^26,28,29,31–33,35^

After the overall visit volume returned to prepandemic levels, telehealth utilization at one health system varied across specialties, ranging from 3% of dermatology visits to 98% of psychiatry visits being delivered via telehealth.^23^ Telehealth was uniformly reported to be used most frequently for behavioral health visits, generally peaking in March–April 2020.^23,30,35,37^ Two studies showed that the total visit volume for behavioral health visits actually increased during the pandemic, especially for mental health, indicating that telehealth use was able to fully offset declines in in-person behavioral health care in these health systems.^30,37^

Similar to results from other types of studies, disparities were found in telehealth utilization related to urbanicity, race/ethnicity, primary language, health insurance status, income, access to other resources, and age.^23–25,29,32,34,38^ Telehealth use was higher for patients who live in urban areas,^25^ are white,^23–25,32,34^ speak English as their first language,^24,32,34^ have health insurance,^25^ have higher incomes,^24^ live in areas with greater access to broadband,^34^ have greater disease burdens or comorbidities,^32,38^ and for young and middle-aged adults compared with children and older adults.^23–25,29,32,34^

Study findings were mixed regarding disparities in telehealth use by insurance status, biological sex, and race/ethnicity. Four studies found higher telehealth utilization among patients with private insurance relative to public insurance,^23,24,32,38^ whereas one study found the opposite.^25^ Two studies found higher telehealth use among men relative to women,^24,32^ and one study found the opposite.^23^ Five studies found higher telehealth utilization among white patients and lower utilization among Latinx and black patients,^23–25,32,34^ and one study found the opposite.^38^ In addition, three studies showed higher telehealth use among Asian patients,^32,34,38^ whereas two studies showed lower telehealth use among Asian patients.^24,25^ One study found higher telehealth use among Native American patients.^25^

One study found no significant differences in patient demographics during the pandemic compared with before the pandemic, indicating that the disparities in telehealth use for this health system are due to preexisting disparities in health care rather than disparities in telehealth, specifically.^23^ Other studies examining disparities in telehealth utilization did not compare differences in patient demographics from before and during the pandemic.

### Other notable studies

We examined seven other notable studies that did not fully fit the selection criteria but still had valuable and relevant information. Chao et al., Patt et al., Portney et al., and Xu et al. focus on specific specialties (surgery, cancer care, ophthalmology, and vitreoretinal care, respectively) and are included here because they nevertheless offer valuable information regarding telehealth utilization across multiple payers and health systems.^39–42^ Demeke et al. and Uscher-Pines et al. provide useful information regarding telehealth utilization within federally qualified health centers (FQHCs).^43–45^

Similar to results from other types of studies, these studies found that in-person visits decreased dramatically during the pandemic while telehealth utilization significantly increased.^39–43,45^ Uscher-Pines et al. found a higher audio visit utilization relative to video visits, and the other studies did not examine differences in telehealth modality.^45^

Telehealth use varied by specialty and health care setting. Uscher-Pines et al. found that FQHCs in California had especially high telehealth utilization for behavioral health services.^45^ Several specialty-specific studies showed very low rates of telehealth utilization during the pandemic, likely due to the necessity of in-person examinations and/or the use of specialized equipment. For example, Xu et al. indicate that there were no recorded retinal telehealth visits before the pandemic, and retinal telehealth utilization remained low over the course of the pandemic, with only 75 visits being recorded per week across the United States.^42^

Portney et al. found that telehealth use increased for ophthalmology, but also remained low compared with other specialties, peaking at 17% of ophthalmic visits.^41^ Similarly, telehealth was only able to slightly offset decreases in surgical visits and cancer-related E&M visits during the pandemic.^39,40^ Patt et al. indicate that from April to July 2020, about 95% of E&M services by providers in professional care settings were delivered via telehealth, whereas telehealth utilization was very low among providers in institutional care settings.^40^

Demeke et al. indicate that telehealth utilization varied by urbanicity and geographic region among FQHCs.^43,44^ Most FQHCs provided telehealth services during the pandemic, with urban FQHCs delivering a greater percentage of their services via telehealth compared with rural FQHCs.^43,44^ Similar to findings from Patel et al. that are described in the *Large-Scale Multipayer, Multihealth System Data Sets* section, Demeke et al. found that FQHCs in the South had the lowest proportion of visits delivered via telehealth.^12,44^

## Discussion

A number of themes and observations can be gleaned from these reports. Across the board, studies reported that in-person visits declined during the early months of the COVID-19 pandemic, while telehealth visits increased dramatically. Several reports indicate that the total primary care visits (both virtual and in-person) dropped by as much as 50% early in the pandemic, and then rebounded over the course of the rest of the year to roughly prepandemic levels. At peak utilization, various forms of telehealth accounted for roughly 15–50% of the total visits in many data sets.

Audio (telephone) visits were widely used and most reports that differentiated between the two visit modalities found that audio was used more often than video visits overall. More sophisticated research comparing these two modalities is certainly warranted. It appears likely that audio telehealth sometimes functions as a technical fall-back when video is not possible or proves problematic, but it may also be a preferred option for some patients or providers. Reasons for this preference and ways of modifying it are worth exploring.

Several reports found that telehealth utilization varied across geographic regions, but results conflicted regarding which regions utilized more or fewer telehealth services. It seems likely that the variability is more accurately attributed to state-specific telehealth policies, either during the pandemic (affecting providers' understanding of the rapidly changing policy environment) or before the pandemic (affecting the latent capacity of providers to quickly adopt telehealth services when demanded). Regardless of the reasons for the variability, it seems of minimal use to look at regional variation in telehealth when state-level data are available and more tightly coupled to potential explanatory factors.

Telehealth use also varied widely across specialties and diagnoses, with behavioral health showing the highest levels of telehealth utilization. This is likely due to several different factors, such as behavioral health being so well suited for telehealth compared with most other specialties. A recent review highlighted several relevant reasons for why behavioral health was likely able to scale telehealth services more rapidly and easily during the pandemic compared with other specialties.^46^ For example, there is a much greater volume of prepandemic published research on telehealth outcomes for mental health relative to other specialties.

In addition, telemental health has received more support from hospitals, health systems, and professional organizations relative to other specialties, and payers have historically been more likely to cover telemental health services. These factors have led to relatively high prepandemic telemental health utilization and engagement, as well as a more well-developed prepandemic telehealth infrastructure for mental health compared with other specialties.

Consequently, telehealth was much more likely to fully offset the decline in in-person visits for behavioral health compared with other specialties. In general, the higher the rate of telehealth utilization evident in any data set, the smaller the observed decrease in overall visit volume during the pandemic, clearly demonstrating that telehealth was substituting for in-person access.

Although few of the studies in this review directly assessed or compared the use of various telehealth modalities (video vs. audio vs. store-and-forward) or patient locations (use of the home as an originating site), it appears safe to assert that the vast majority of telehealth visits that occurred during the pandemic would not have been reimbursable without the major telehealth-related policy changes that took place as a result of the PHE, such as allowing reimbursement for audio visits and removing restrictions on the patient's location for telehealth visits.

These policy changes allowed for telehealth services to partially (and sometimes fully) offset the decline in in-person visits, reduce the risk of COVID-19 exposure and transmission, preserve personal protective equipment, and give many patients peace of mind while continuing to access necessary health care services. We hope to further explore the impact of telehealth-related policy changes on telehealth utilization during the COVID-19 pandemic in a future study.

Despite these many benefits, disparities in access to telehealth were found. Overall, telehealth utilization was higher among patients in urban areas, areas with greater broadband availability, and areas with higher prepandemic levels of telehealth utilization. Telehealth use was also generally higher among patients who were white, spoke English as their first language, had health insurance (especially private insurance), had higher incomes, had greater disease burdens, and were middle aged (compared with children and older adults). Results regarding telehealth utilization by biological sex were mixed.

Most studies specifically examining disparities in telehealth utilization did not compare differences in patient demographics from before and during the pandemic, but the one study that did found no significant differences in the demographic composition of these two groups of patients.^23^ An analysis by Poeran et al. demonstrated that disparities in telehealth utilization existed before the COVID-19 pandemic and were gradually worsening over time, with older patients, patients with more comorbidities, patients living in rural areas, and patients with lower incomes having lower rates of telehealth utilization.^47^

Taken together, this suggests that disparities in telehealth use and access reported in the current studies reflect characteristics of health care and society more generally rather than specific characteristics of telehealth. It is reasonable to predict that telehealth by itself will not mitigate these preexisting disparities or expand access to health care absent contextual drivers (policies and programs) specifically designed to do so.

In addition, access to many services and specialties is already limited by factors such as workforce shortages, poor access to health insurance, poor broadband availability, and discomfort and unfamiliarity with navigating the health care system. To eliminate disparities in telehealth utilization and access, the underlying structural inequities in access to both health care services and technology will need to be addressed.

## Conclusion and Limitations

Almost all of the large-scale, multipayer, multihealth system data set analyses we reviewed used encounter data from commercially insured individuals (and Medicare Advantage plans). There was markedly less representation of patients with public (Medicare, Medicaid) insurance, or the uninsured. More research on telehealth utilization trends during the pandemic among publicly insured populations will help to more fully describe the impact of the PHE-related telehealth policies, especially for underserved populations that are not commercially insured.

Furthermore, each of these studies used a data set that is a universe of its own, subject to many factors apart from those brought about by the pandemic. All are influenced by the (shifting) policies driving utilization, and each contains data that are heterogeneous on multiple levels. Even though averages can be derived, the usefulness of such averages is suspect. One cannot meaningfully provide an “average” of telehealth use for multiple states or payers with different policies that directly affect utilization.

Despite these limitations, the information provided by these large-scale studies is of immense value because it provides insight regarding the impact of PHE-related telehealth policies on different telehealth modalities, the extent that telehealth was able to offset declines in in-person visits, telehealth use across different specialties and diagnoses, geographical variation in telehealth use, and disparities in telehealth use. This review also forms an appropriate starting point for further examination as more large-scale studies become available.

## Authorship Contribution Statements

J.N. and A.H. conceived of this review article. A.H. performed the literature search, and J.N. verified the search methods and supervised the findings of this work. A.H. and J.N. interpreted the literature review findings to formulate the conclusions. A.H. wrote the first draft of this article, and J.N. revised and expanded on this draft. A.H. and J.N. have reviewed this article, approve of its publication in this journal, and agree to be accountable for all aspects of this work. This article has been submitted solely to Telemedicine Reports and is not published, in press, or submitted elsewhere.

## Supplementary Material

Supplemental data

## Abbreviations Used

ASPE: Assistant Secretary for Planning and Evaluation
EHR: electronic health records
FFS: fee-for-service
FQHCs: federally qualified health centers
HRSA: Health Resources and Services Administration
MEDPAC: Medicare Payment Advisory Commission
PHE: public health emergency
VA: veterans health administration
