# Supplementary material for: Telehealth Utilization During the COVID-19 Pandemic: A Preliminary Selective Review
Source: Telemed Rep. 2022 Feb 3;3(1):38–47. doi: 10.1089/tmr.2021.0040 (PMC8989093; doi:10.1089/tmr.2021.0040)
Supplement: Supplemental data [file Suppl_TableS1.docx]

**Table 1:** Summary of the 39 articles included in this review.

| **Article Citation** | **Population** | **Definition of Telehealth** | **Study Objective(s)** | **Findings** |
| --- | --- | --- | --- | --- |
| **Large-Scale, Multi-Payer, Multi-System Datasets** | | | | |
| Alexander, GC, Tajanlangit, M, Heyward, J, Mansour, O, Qato, DM, & Stafford, RS. Use and Content of Primary Care Office-Based vs Telemedicine Care Visits During the COVID-19 Pandemic in the US. JAMA Netw Open 2020;3(10). DOI: 10.1001/jamanetworkopen.2020.21476 | 125.8 million primary care visits (data obtained from the US National Disease and Therapeutic Index) | Encounters with "telemedicine" listed as the site of service. | Describe changes in primary care services that were delivered in-person and via telehealth during the pandemic with regard to visit volume, type, and content. | During the pandemic, in-person visits declined by 50% while telehealth visits increased from 1% of all visits to 35%. Telehealth utilization was not associated with regional COVID-19 prevalence and was similar among White and Black patients. Telehealth use varied by region, ranging from 15% of visits in the East North Central region to 27% of visits in the Pacific region. |
| Cantor, JH, McBain, RK, Pera, MF, Bravata, DM, & Whaley, CM. Who Is (and Is Not) Receiving Telemedicine Care During the COVID-19 Pandemic. Am J Prev Med 2021;000(000):1-5. DOI: 10.1016/j.amepre.2021.01.030 | 6.8 and 6.4 million employer-based health plan enrollees from 2020 and 2019, respectively (data obtained from Castlight Health) | CPT codes with "telemedicine" listed as the site of service. Includes video visits and audio visits. | Investigate the impact of COVID-19 on disparities in telehealth and in-person health care utilization among commercially insured individuals. | Telehealth use increased more than 20-fold after March 13, 2020, while in-person visits decreased by almost 50% and were not completely counterbalanced by the increase in telehealth use. Telehealth utilization increases were highest in urban areas, counties with lower poverty levels, and among adults. |
| Mehrotra, A, Chernew, M, Linetsky, D, Hatch, H, Cutler, D, & Schneider, EC. The Impact of the COVID-19 Pandemic on Outpatient Care: Visits Return to Prepandemic Levels, but Not for All Providers and Patients. New York, NY: The Commonwealth Fund, 2020. | More than 50,000 providers that are Phreesia clients | Visits with "telemedicine" listed as the appointment type and/or location within the scheduling software. Includes video visits and audio visits. | Describe the impact of the COVID-19 pandemic on health care utilization, telehealth utilization, and health care provider costs. | Organizations with more providers delivered a higher percentage of their visits via telehealth than organizations with fewer providers. About a third of provider organizations never used telehealth during the pandemic, and from April to September 2020 many organizations went from delivering 5% or more of services via telehealth to less than 5%. |
| Mehrotra, A, Chernew, M, Linetsky, D, Hatch, H, Cutler, D, & Schneider, EC. The Impact of COVID-19 on Outpatient Visits in 2020: Visits Remained Stable, Despite a Late Surge in Cases. New York, NY: The Commonwealth Fund, 2021. | More than 50,000 providers that are Phreesia clients | Visits with "telemedicine" listed as the appointment type and/or location within the scheduling software. Includes video visits and audio visits. | Describe the impact of the COVID-19 pandemic on health care utilization, telehealth utilization, and health care provider costs. | Telehealth use peaked in April 2020 then slowly declined through October 2020 (still remaining much higher than the pre-pandemic baseline), then increased again in November and December 2020. Telehealth utilization in December 2020 varied by specialty, ranging from 0% of ophthalmology visits to 56% of behavioral health visits. |
| Patel, SY, Rose, S, Barnett, ML, Huskamp, HA, Uscher-Pines, L, & Mehrotra, A. Community Factors Associated With Telemedicine Use During the COVID-19 Pandemic. JAMA Netw Open 2021;4(5). DOI: 10.1001/jamanetworkopen.2021.10330 | 2,800 counties with at least 100 Medicare Advantage enrollees (data obtained from OptumLabs Data Warehouse) | CPT codes with GT, GQ, or 95 modifiers (video visits) and CPT codes 99441 - 99443 (audio visits). | Investigate the association of telehealth utilization with several community factors among commercially insured and Medicare Advantage enrollees during the pandemic. | The median percentage of visits occurring via telehealth was 19% (ranging from 10% to 31%). Counties with lower incomes, population density, broadband availability, pre-pandemic telehealth utilization, and COVID-19 cases per capita at the beginning of the pandemic tended to have lower telehealth utilization during the pandemic. |
| Patel, SY, Mehrotra, A, Huskamp, HA, Uscher-Pines, L, Ganguli, I, & Barnett, ML. Trends in Outpatient Care Delivery and Telemedicine During the COVID-19 Pandemic in the US. JAMA Intern Med 2021;181(3). DOI: 10.1001/jamainternmed.2020.5928 | 16.7 million commercially insured and Medicare Advantage enrollees (data obtained from OptumLabs Data Warehouse) | CPT codes with GT, GQ, or 95 modifiers (video visits) and CPT codes 99441 - 99443 (audio visits). | Analyze outpatient visits that occurred in-person and via telehealth to investigate how telehealth offset the decrease in outpatient visits during the pandemic among commercially insured and Medicare Advantage enrollees. | From January to June 2020, telehealth visits increased from 1 to 18 per 1,000 enrollees (peaking in April 2020), whereas in-person visits decreased from 103 to 76 and total visits declined from 104 to 94. By May - June 2020, the percentage of total visits that occurred via telehealth ranged from 8% in South Dakota to 48% in Massachusetts. States in the South tended to have lower telehealth utilization. |
| Patel, SY, Mehrotra, A, Huskamp, HA, Uscher-Pines, L, Ganguli, I, & Barnett, ML. Variation In Telemedicine Use And Outpatient Care During The COVID-19 Pandemic In The United States. Health Affairs 2021;40(2). DOI: 10.1377/hlthaff.2020.01786 | 16.7 million commercially insured and Medicare Advantage enrollees (data obtained from OptumLabs Data Warehouse) | CPT codes with GT, GQ, or 95 modifiers (video visits) and CPT codes 99441 - 99443 (audio visits). | Describe changes in outpatient visits and telehealth utilization across patient demographics, diagnoses, and provider specialties among commercially insured and Medicare Advantage enrollees from January - June 2020. | During the pandemic, 30% of visits were delivered via telehealth, and weekly telehealth visits increased 23-fold compared to pre-pandemic. Telehealth use was inversely associated with poverty rates. Telehealth use varied by specialty, ranging from 9% of ophthalmologists using any telehealth to 68% of endocrinologists. Telehealth use also varied by diagnosis, ranging from 3% of visits for glaucoma to 53% of visits for depression. Higher telehealth use was associated with smaller declines in total visits. |
| Weiner, JP, Bandeian, S, Hatef, E, Lans, D, Liu, A, & Lemke, KW. In-Person and Telehealth Ambulatory Contacts and Costs in a Large US Insured Cohort Before and During the COVID-19 Pandemic. JAMA Netw Open 2021;4(3). DOI: 10.1001/jamanetworkopen.2021.2618 | 36.6 million patients (data obtained from Blue Health Intelligence) | CPT codes with GT, GQ, or 95 modifiers; "02" (telehealth) as the place of service code; and specific CPT codes that are classified as telehealth only. Includes video visits, audio visits, and other telehealth modalities. | Describe changes in in-person and telehealth ambulatory care visits during the COVID-19 pandemic. | Ambulatory care visits decreased by 18% from 2019 to 2020 and telehealth utilization increased from 0.3% of all visits in 2019 to 24% of all visits in 2020. Higher telehealth utilization was associated with higher disease burden, COVID-19 prevalence, and more social resources. |
| Whaley, CM, Pera, MF, Cantor, J, et al. Changes in Health Services Use Among Commercially Insured US Populations During the COVID-19 Pandemic. JAMA Netw Open 2020;3(11). DOI: 10.1001/jamanetworkopen.2020.24984 | 5.6 - 6.8 million commercially insured individuals from 2018 - 2020 (data obtained from employer-based health plan claims) | CPT codes with GT, GQ, or 95 modifiers; "02" (telehealth) as the place of service code; and CPT codes 99441-99444, 99421-99423, 98970-98972, G2061-G2063. Includes video visits and audio visits. | Describe changes in health care utilization among commercially insured individuals during the beginning of the COVID-19 pandemic. | In March and April 2020, preventative and elective care utilization significantly declined while telehealth utilization increased, although not enough to compensate for the declines in in-person care. These changes in in-person and telehealth service utilization exhibited disparities with regard to income and race/ethnicity. |
| Ziedan, E, Simon, KI, & Wing, C. Effects of State COVID-19 Closure Policy on Non-COVID-19 Health Care Utilization (Working Paper). Cambridge, MA: National Bureau of Economic Research, 2020. DOI: 10.3386/w27621 | 35 million patients (data obtained from Healthjump) | CPT codes that are classified as telehealth only or telehealth eligible (non telehealth eligible codes are classified as face to face visits). | Describe how the COVID-19 pandemic has impacted non-COVID-19-related acute care and primary care visits. | Outpatient visits declined by 15-16% within two weeks of state closure policies and these visits started to rebound in mid-April, though usually not to pre-pandemic levels. Mental health outpatient visits declined less than other types of outpatient visits, likely due to the rapid transition of mental health services to telehealth. |
| **Limited Datasets** | | | | |
| Single Payer Data | | | | |
| Bosworth, A, Ruhter, J, Samson, LW, et al. Medicare Beneficiary Use of Telehealth Visits: Early Data from the Start of COVID-19 Pandemic. Washington, DC: Office of the Assistant Secretary for Planning and Evaluation, U.S. Department of Health and Human Services, 2020. | 38.5 million Medicare fee-for-service Part B beneficiaries | Synchronous and asynchronous patient-to-provider communication. Includes video visits, audio visits, chat, and email. | Describe Medicare fee-for-service primary care telehealth utilization during the beginning of the COVID-19 pandemic. | The proportion of Medicare primary care visits that were delivered via telehealth increased from 0.1% in February 2020 to 44% in April 2020. Telehealth utilization declined as in-person visits rebounded in mid-April and May, then leveled off by June. Urban areas tended to have larger increases in Medicare primary care telehealth utilization compared to rural areas. |
| Koonin, LM, Hoots, B, Tsang, CA, et al. Trends in the Use of Telehealth During the Emergence of the COVID-19 Pandemic — United States, January–March 2020. MMWR Morb Mortal Wkly Rep 2020;69(43). DOI: 10.15585/mmwr.mm6943a3 | 2.7 million encounters from Amwell, Teladoc, MDLIVE, and Doctor on Demand | All encounters from four major telehealth providers. | Describe changes in telehealth utilization during the beginning of the pandemic. | Telehealth utilization increased by 50% from the first quarter of 2019 to the first quarter of 2020, peaking at a 154% increase in visits at the end of March 2020. The portion of visits that were related to COVID-19 significantly increased from 6% to 16% in March 2020. |
| Medicare Payment Advisory Commission (MEDPAC). Report to the Congress: Medicare Payment Policy. Washington, D.C.: MEDPAC, 2021. | 10.3 million Medicare fee-for-service beneficiaries who received at least one telehealth service | Telehealth services that are paid under Medicare's physician fee schedule. Includes video visits and audio visits. | Describe Medicare fee-for-service telehealth utilization during COVID-19. | From January to June 2020, about one third of Medicare fee-for-service (FFS) beneficiaries had at least one telehealth encounter. Telehealth encounters increased from 102,000 in February to 8.4 million in April, then decreased to 5.6 million in June. During this time, 78% of Medicare FFS telehealth encounters were for primary care visits, and about one third of these visits were audio evaluation and management services. |
| VA Electronic Health Records (EHR) Data | | | | |
| Cornwell, BL, Szymanski, BR, & McCarthy, JF. Impact of the COVID-19 Pandemic on Primary Care–Mental Health Integration Services in the VA Health System. Psychiatr Serv (ahead of print) 2021. DOI: 10.1176/appi.ps.202000607 | Patient encounters from the Veterans Health Administration | Synchronous audio and video visits with the patient either at home or in the clinic. | Examine weekly trends in individual and group primary care-mental health integration (PCMHI) encounters that occurred in-person, over the phone, and via telehealth (with the patient at home or onsite) during the pandemic. | From March to July 2020, in-person visits decreased by 90%, audio visits increased by 147%, and home video telehealth increased by 645% for PCMHI encounters. Utilization recovered by 15% after March 2020 and remained at about 82% of pre-pandemic levels after that. Sites that offered telehealth services in December 2019 had an 11% increase in visits from March to July 2020, whereas sites that didn't experienced a 10% reduction in services. |
| Ferguson, JM, Jacobs, J, Yefimova, M, Greene, L, Heyworth, L, & Zulman, DM. Virtual care expansion in the Veterans Health Administration during the COVID-19 pandemic: clinical services and patient characteristics associated with utilization. J Am Med Inform Assoc 2021;28(3). DOI: 10.1093/jamia/ocaa284 | 43 million patient encounters from the Veterans Health Administration Corporate Data Warehouse | Encounters with VA Managerial Cost Accounting Stop Codes that are classified as virtual care (video and audio visits) or remote patient monitoring. | Describe the transition from in-person to telehealth service delivery within Veterans Affairs at the beginning of the pandemic and identify patient populations that need additional resources for overcoming barriers to accessing telehealth services. | Before the pandemic, 14% of VA visits were delivered virtually, compared to 58% of visits by June 2020. During the pandemic, telehealth utilization was higher among veterans who weren't unhoused, lived in urban areas, were aged 18-44 (compared to 45+), and who had lower incomes, higher disability, and more chronic conditions. |
| Spelman, JF, Brienza, R, Walsh, RF, et al. A Model for Rapid Transition to Virtual Care, VA Connecticut Primary Care Response to COVID-19. J Gen Intern Med 2020;35(10). DOI: 10.1007/s11606-020-06041-4 | Over 58,000 veterans from the VA Connecticut Health Care System | Video visits, audio visits, and secure messaging. | Describe a large primary care system's approach for rapidly transitioning to telehealth during the pandemic and the resulting outcomes relating to encounter types and respiratory urgent care utilization. | Most primary care visits were transitioned from in-person to telehealth in less than two weeks. Most telehealth visits were for audio encounters, but video visits also increased almost 18-fold. |
| Single Health System Electronic Health Records (EHR) Data | | | | |
| Drake, C, Lian, T, Cameron, B, Medynskaya, K, Bosworth, HB, & Shah, K. Understanding Telemedicine's "New Normal": Variations in Telemedicine Use by Specialty Line and Patient Demographics. Telemed J E Health (ahead of print) 2021. DOI: 10.1089/tmj.2021.0041 | 239,803 patients, 245,648 patients, and 624,886 patients at Duke University Health System | Video and audio visits. | Assess differences in telehealth use by specialty and patient demographics during the COVID-19 pandemic after in-person visits resumed and overall visit volume went back to pre-pandemic levels. | Telehealth utilization varied across specialties, ranging from 3% of dermatology visits to 98% of psychiatry visits being delivered via telehealth. Telehealth utilization was lower among Black patients, men, older patients, and patients with public insurance, relative to White patients, women, younger patients, and patients with commercial insurance. There were no significant differences in patient demographics during the pandemic compared to before the pandemic. |
| Eberly, LA, Kallan, MJ, Julien, HM, et al. Patient Characteristics Associated With Telemedicine Access for Primary and Specialty Ambulatory Care During the COVID-19 Pandemic. JAMA Netw Open 2020;3(12). DOI: 10.1001/jamanetworkopen.2020.31640 | 148,402 patients at a large academic health system | Video and audio visits. | Assess which patient demographics are associated with higher telehealth utilization, especially for video visits, for primary care and specialty ambulatory care during the beginning of the COVID-19 pandemic. | During the beginning of the pandemic, telehealth utilization for primary care and specialty ambulatory care was lower for older patients, Asian patients, patients who didn't speak English as their first language, and Medicaid enrollees. Video-based telehealth utilization was lower for older patients, women, Black and Latinx patients, and patients with lower household incomes. |
| Hsiao, V, Chandereng, T, Lankton, RL, et al. Disparities in Telemedicine Access: A Cross-Sectional Study of a Newly Established Infrastructure during the COVID-19 Pandemic. Appl Clin Inform (ahead of print) 2021. DOI: 10.1055/s-0041-1730026 | 197,076 patients at an integrated academic health system | Video and audio visits. | Assess telehealth utilization trends and identify possible disparities worsened by increased telehealth use during the COVID-19 pandemic. | Video visit utilization was lower among older adults; those who live in rural areas; Asian, Black, and Latinx patients; and uninsured patients. Video visit utilization was higher among Medicaid and Medicare patients, Native American patients, and patients with higher digital literacy. |
| Kazi, R, Evankovich, MR, Liu, R, et al. Utilization of Asynchronous and Synchronous Teledermatology in a Large Health Care System During the COVID-19 Pandemic. Telemed J E Health 2021;27(7). DOI: 10.1089/tmj.2020.0299 | 2,632 teledermatology visits / 2,530 patients at University of Pittsburgh Medical Center Health System | Synchronous video and audio visits, and asynchronous store-and-forward visits. | Describe telehealth utilization trends for dermatology during the pandemic. | Of all teledermatology visits, 36% were asynchronous and 64% were synchronous. Less than 5% of visits needed an in-person follow-up. Prescriptions for antibiotics and non-retinoid acne medications were more common in asynchronous visits, and prescriptions for immunomodulators and biologics were more common in synchronous visits. |
| Leyton, C, Zhang, C, & Rikin, S. Evaluation of the Effects of the COVID-19 Pandemic on Electronic Consultation Use in Primary Care. Telemed J E Health (ahead of print) 2021. DOI: 10.1089/tmj.2020.0547 | 193,263 primary care appointments at Montefiore Medical Center | Virtual visits (patient-to-provider video and audio visits) and e-consults (electronic communication between a primary care provider and a specialist). | Assess changes in e-consult utilization among primary care providers and in specialists' suggestions for specialty visits during the COVID-19 pandemic. | During the pandemic, the odds of primary care providers initiating e-consults and the odds of specialists recommending specialty appointments both increased. Primary care providers initiated 1,318 e-consults with specialists during 193,263 primary care appointments during the pandemic. |
| Madden, N, Emeruwa, UN, Friedman, AM, et al. Telehealth Uptake into Prenatal Care and Provider Attitudes during the COVID-19 Pandemic in New York City: A Quantitative and Qualitative Analysis. Am J Perinatol 2020;37(10). DOI: 10.1055/s-0040-1712939 | 4,248 pregnancy related ambulatory visits at Columbia University Irving Medical Center-affiliated prenatal practices | Mostly video visits, with some audio visits in cases where technical and/or broadband issues prevented the use of video. | Assess the extent that prenatal care services transitioned to telehealth during the COVID-19 pandemic. | From March 9 to April 12, 2020, 32% of pregnancy related ambulatory visits were delivered via telehealth. By April 12, 56% of generalist obstetrics and gynecology visits, 62% of maternal-fetal medicine visits, and 42% of clinic visits were delivered via telehealth. |
| Mann, DM, Chen, J, Chunara, R, Testa, PA, & Nov, O. COVID-19 transforms health care through telemedicine: Evidence from the field. J Am Med Inform Assoc 2020;27(7). DOI: 10.1093/jamia/ocaa072 | 7.5 million active patients at NYU Langone Health | Video visits. | Describe the feasibility of telehealth use and its impact on urgent and nonurgent health care services in a large health system during the COVID-19 pandemic. | Telehealth utilization for urgent care increased from 102 visits per day to 802 (a 683% increase) between March 2 and April 14, 2020. Of all telehealth visits after the expansion of telehealth services, 56% and 18% of urgent care and non-urgent care visits, respectively, were related to COVID-19. The age group with the highest telehealth utilization was patients 20 to 44 years old, especially for urgent care. |
| Miller, MJ, Watson, ES, Horberg, MA, Bhatia, M, Tripuraneni, BR, & McCarthy, RJ. Patient experience After Modifying Visit Delivery During the COVID-19 Pandemic. Am J Manag Care 2021;27(2). DOI: 10.37765/ajmc.2021.88535 | 2.2 million medical visits from Kaiser Permanente Mid-Atlantic States | Video and audio visits. | Assess changes in health care visits, visit modality, and patient experience during the COVID-19 pandemic. | Between March and May 2020 average weekly overall visits decreased from 54,479 to 12,848, dropping further to 10,247 between May and June 2020. During this time, audio visits increased from 7,897 to 27,177 and 25,018 and video visits increased from 1,039 to 9,292 and 20,158. |
| Neeman, E, Kolevska, T, Reed, M, et al. Cancer Care Telehealth Utilization Rates and Provider Attitudes in the Wake of the Novel Coronavirus Pandemic: The Kaiser Permanente Northern California Experience. Clinical Cancer Research 2020;26(18). DOI: 10.1158/1557-3265.COVID-19-S06-03 | 104,588 oncology visits at Kaiser Permanente Northern California | Video and audio visits. | Describe in-person, audio, and video visit utilization trends for cancer care before and after California established shelter-in-place (SIP) orders on March 19, 2020. | After SIP orders went into effect, in-person visits deliced from 55% to 3% while audio visits increased from 44% to 79% and video visits increased from 0.5% to 18%. From December 2019 to May 2020, video visits increased from 0.4% of all visits to 31%. Audio visits peaked at 87% of visits in April 2020, while in-person visits were at a low of 2%. |
| Neeman, E, Lyon, L, Sun, H, et al. The Future of Tele-Oncology: Trends and Disparities in Telehealth and Secure Message Utilization in the COVID-19 Era. Am J Clin Oncol 2021;39. DOI: 10.1200/JCO.2021.39.15_suppl.1506 | 334,666 visits and 1.2 million secure messages at Kaiser Permanente Northern California Hematology and Oncology practices | Video visits, audio visits, and secure messaging. | Describe disparities and utilization trends for telehealth and secure messaging (SM) at a large integrated health system before and during the COVID-19 pandemic. | During the pandemic, the monthly average of overall visits decreased only by 4% due to the increase in telehealth utilization. In-person visits decreased from 11,001 to 2,170 (-80%) while video visits increased from 40 to 4,666 (11,565%) and audio visits increased from 5,114 to 8,663 (69%). Telehealth utilization was higher among younger patients, commercially insured patients, patients who speak English as their first language, Asian and White patients, men, patients who are married, and patients with greater disease burdens. |
| Patt, D, Wilfong, L, Kanipe, K, & Paulson, RS. Telemedicine for Cancer Care: Implementation Across a Multicenter Community Oncology Practice. Am J Manag Care 2020a;26(N Spec No.). DOI: 10.37765/ajmc.2020.88560 | 221 sites of service and more than 650 practitioners at Texas Oncology | Mostly video visits, with some audio visits in cases where technical and/or broadband issues prevented the use of video. | Assess telehealth utilization in a statewide oncology practice during the COVID-19 pandemic. | From April to October 2020, 15-20% of new patient visits and 20-25% of established patient visits were delivered via telehealth. Almost all (96%) of the providers utilized telehealth, with 33% using telehealth for more than 25% of visits. Most of these telehealth encounters are video visits, and fewer than 5% of providers indicate regularly using audio visits. |
| Rodriguez, JA, Betancourt, JR, Sequist, TD, & Ganguli, I. Differences in the Use of Telephone and Video Telemedicine Visits During the COVID-19 Pandemic. Am J Manag Care 2021;27(1). DOI: 10.37765/ajmc.2021.88573 | 231,596 visits by 162,102 patients at Mass General Brigham | CPT codes with GT modifiers (video visits) or GPH modifiers (audio visits). | Describe patient and neighborhood characteristics that are associated with in-person, audio, and video visit utilization, and describe patient, physician, and practice factors that influence decisions regarding visit modality during the COVID-19 pandemic. | Of the 231,596 encounters, 32% were audio visits and 34% were video visits. Patients were less likely to use telehealth services if they were older than 65, Latinx, Spanish-speaking, or from areas with lower access to broadband. |
| Uscher-Pines, L, Thompson, J, Taylor, P, et al. Where Virtual Care Was Already a Reality: Experiences of a Nationwide Telehealth Service Provider During the COVID-19 Pandemic. J Med Internet Res 2020;22(12). DOI: 10.2196/22727 | Doctor on Demand patients | All encounters from a major telehealth provider. | Assess telehealth use and changes in visit volume, reasons for visits, and patients served by Doctor On Demand before and during the COVID-19 pandemic. | Total visit volume increased to 59% above baseline in March-April 2020 then decreased to 15% above baseline in June 2020. Increases in visit volume peaked at 64% above baseline for rural patients and 58% for urban patients. Unscheduled visits for behavioral health and chronic illness increased through April 2020 to 109% and 131% above baseline, respectively, then declined through June 2020 to 69% and 37% above baseline, respectively. |
| Xu, S, Glenn, S, Sy, L, et al. Impact of the COVID-19 Pandemic on Health Care Utilization in a Large Integrated Health Care System: Retrospective Cohort Study. J Med Internet Res 2021;23(4). DOI: 10.2196/26558 | 4.5 - 4.6 million members at Kaiser Permanente Southern California in 2019 and 2020 | Video and audio visits. | Assess changes in in-person and telehealth service utilization during the COVID-19 pandemic and compare utilization from before and during the pandemic. | During the beginning of the pandemic, inpatient, ED, and outpatient visits decreased by 30%, 37%, and 81%, respectively, while telehealth utilization increased 4-fold. By the end of June 2020, the increase in telehealth utilization compensated for the decline in outpatient visits. |
| Yang, J, Landrum, MB, Zhou, L, & Busch, AB. Disparities in Outpatient Visits for Mental Health and/or Substance Use Disorders During the COVID Surge and Partial Reopening in Massachusetts. Gen Hosp Psychiatry 2020;67. DOI: 10.1016/j.genhosppsych.2020.09.004 | 52,907 - 73,184 patients per year at Mass General Brigham | Synchronous video and audio visits, and asynchronous e-visits and e-consults. | Describe changes in outpatient behavioral health visits by diagnosis, provider type, patient race/ethnicity, insurance, and visit type (telehealth vs. in-person) during the first peak in COVID-19 cases and partial state reopening in Massachusetts. | About 5% of behavioral health visits were delivered via telehealth prior to the pandemic, compared to 83-84% after the first spike in COVID-19 cases. During this surge, non-behavioral health visits decreased by 38% and behavioral health visits increased by 9%. During the partial state reopening, mental health visits went back to pre-pandemic volume while SUD visits decreased by 31%. |
| Yuan, N, Pevnick, JM, Botting, PG, et al. Patient Use and Clinical Practice Patterns of Remote Cardiology Clinic Visits in the Era of COVID-19. JAMA Netw Open 2021;4(4). DOI: 10.1001/jamanetworkopen.2021.4157 | 176,781 ambulatory cardiology visits from an urban, multisite health system | Video and audio visits. | Assess potential association between the shift to remote cardiology ambulatory visits and disparities in access to health care, diagnostic testing, and medication prescribing during the COVID-19 pandemic. | During the COVID-19 pandemic, telehealth utilization was higher among Asian, Black, and Latinx patients; patients with private insurance; and patients with cardiovascular comorbidities. Health care providers were less likely to order medication or tests during telehealth visits that occurred during the pandemic compared to in-person visits that occurred before the pandemic. |
| **Other Notable Studies** | | | | |
| Chao, GF, Li, KY, Zhu, Z, et al. Use of Telehealth by Surgical Specialties During the COVID-19 Pandemic. JAMA Surg 2021;156(7). DOI: 10.1001/jamasurg.2021.0979 | 4,405 surgeons (data obtained from a Michigan statewide commercial payer) | Video and audio visits. | Describe telehealth utilization trends across surgical specialties before and during the COVID-19 pandemic. | Telehealth utilization increased during the beginning of the pandemic and declined later in the pandemic, differing by surgical specialty. Overall, telehealth was able to offset only a small percentage of 2020 surgical visits relative to 2019. |
| Demeke, HB, Merali, S, Marks, S, et al. Trends in Use of Telehealth Among Health Centers During the COVID-19 Pandemic — United States, June 26–November 6, 2020. MMWR Morb Mortal Wkly Rep 2021;70(7). DOI: 10.15585/mmwr.mm7007a3 | 245 health centers that completed a weekly HRSA Health Center COVID-19 Survey | Not specified. | Describe trends in health center telehealth utilization and visit volume in the United States from June 26 - November 6, 2020. | Telehealth visits decreased as COVID-19 cases decreased but stayed constant as COVID-19 cases increased. Overall, 30% of health center visits were via telehealth, and health centers in the South and rural areas consistently had the lowest portion of visits delivered via telehealth. |
| Demeke, HB, Pao, LZ, Clark, H, et al. Telehealth Practice Among Health Centers During the COVID-19 Pandemic — United States, July 11–17, 2020. MMWR Morb Mortal Wkly Rep 2020;69(50). DOI: 10.15585/mmwr.mm6950a4 | 1,009 health centers that completed a weekly HRSA Health Center COVID-19 Survey | Not specified. | Describe trends in health center telehealth utilization and visit volume in the United States for the week of July 11-17, 2020. | Overall, 95% of health centers provided telehealth services. Urban health centers were more likely than rural health centers to provide more than 30% of their visits via telehealth. |
| Patt, D, Gordan, L, Diaz, M, et al. Impact of COVID-19 on Cancer Care: How the Pandemic Is Delaying Cancer Diagnosis and Treatment for American Seniors. JCO Clin Cancer Inform 2020b;4. DOI: 10.1200/CCI.20.00134 | 5%-7% of the Medicare FFS population / 6.2 million Medicare FFS claims (data obtained from a large medical claims clearinghouse database) | Visit modality was determined using claim type (as opposed to place of service code), but how claim types were defined and categorized was not specified. | Document reductions in cancer screenings and treatment throughout the United States during the COVID-19 pandemic. | There was a decrease in cancer screenings, visits, therapy, and surgeries in March-July 2020 compared with March-July 2019, with variation by cancer type and service site. Telehealth use was able to slightly offset decreases in cancer-related Evaluation & Management (E&M) visits, from -73% to -58%. From April-July 2020, about 95% of E&M services by providers in professional care settings were delivered via telehealth, whereas telehealth use was very low among providers in institutional care settings. |
| Portney, DS, Zhu, Z, Chen, EM, et al. COVID-19 and Use of Teleophthalmology (CUT Group): Trends and Diagnoses. Ophthalmology (ahead of print) 2021. DOI: 10.1016/j.ophtha.2021.02.010 | 362,355 ophthalmology visits (data obtained from Blue Cross Blue Shield of Michigan) | CPT codes with GT or “25” modifiers, and CPT codes 92227 and 92228 (store-and-forward retinal imaging codes). [Authors may have intended “95” -AH/JN] | Describe the characteristics of ophthalmology telehealth utilization before and during the COVID-19 pandemic. | Prior to the pandemic 0.04% of ophthalmic visits occurred via telehealth, which increased to 1.6% from March 15, 2020 - September 1, 2020. During the peak of the pandemic, 17% of ophthalmic visits occurred via telehealth. About 30% of ophthalmologists used telehealth prior to the pandemic and this number rose to 37% by September 2020. |
| Uscher-Pines, L, Sousa, J, Jones, M, et al. Telehealth Use Among Safety-Net Organizations in California During the COVID-19 Pandemic. JAMA 2021;325(11). DOI: 10.1001/jama.2021.0282 | 41 California FQHCs with 534 locations (data obtained from HRSA’s Uniform Data System) | Video and audio visits. | Describe trends in audio, video, and in-person visits among California FQHCs before and during the COVID-19 pandemic. | There was minimal telehealth use among California FQHCs before the pandemic, but during the pandemic 48% of visits were in-person, 49% were audio visits, and 3% were video visits. For behavioral health, 23% of visits were in-person, 63% were audio visits, and 14% were video visits. |
| Xu, D, Starr, MR, Boucher, N, et al. Real-world vitreoretinal practice patterns during the 2020 COVID-19 pandemic: a nationwide, aggregated health record analysis. Curr Opin Ophthalmol 2020;31(5). DOI: 10.1097/ICU.0000000000000692 | 1.6 million patients / 11 million vitreoretinal encounters (data obtained from Vestrum Health, LLC) | Not specified, but likely all or mostly video visits. | Describe changes in vitreoretinal specialist office visits, telehealth use, and intravitreal injection volume before and during the COVID-19 pandemic. | Before the pandemic there were an average of 50,061 weekly vitreoretinal office visits, which decreased to 32,814 during the pandemic. Before the pandemic there were no recorded retinal telehealth visits, and retinal telehealth utilization remained low over the course of the pandemic with only 75 retinal telehealth visits being recorded per week across the United States. |
